# Intrinsic Auricular Muscle Zone Stimulation Improves Walking Parameters of Parkinson's Patients Faster Than Levodopa in the Motion Capture Analysis: A Pilot Study

**DOI:** 10.3389/fneur.2020.546123

**Published:** 2020-10-07

**Authors:** Yusuf O. Cakmak, Burak Ozsoy, Sibel Ertan, Ozgur O. Cakmak, Gunes Kiziltan, Hale Yapici-Eser, Ecem Ozyaprak, Selim Olcer, Hakan Urey, Yasemin Gursoy-Ozdemir

**Affiliations:** ^1^Cakmak Lab, Department of Anatomy, School of Biomedical Sciences, University of Otago, Dunedin, New Zealand; ^2^Centre for Health Systems and Technology, Dunedin, New Zealand; ^3^Medical Technologies Centre of Research Excellence, Auckland, New Zealand; ^4^Brain Health Research Centre, Dunedin, New Zealand; ^5^Koc University & Koc Holding's Early-Stage Technology, Innovation and IP Investment, Commercialization, and Advisory Company (Inventram), Istanbul, Turkey; ^6^Department of Neurology, School of Medicine, Koc University Research Centre for Translational Medicine (KUTTAM), Koç University, Istanbul, Turkey; ^7^Department of Neurology, Cerrahpaşa School of Medicine, Istanbul University, Istanbul, Turkey; ^8^Department of Psychiatry, School of Medicine, Koç University, Istanbul, Turkey; ^9^Department of Electrical Engineering, College of Engineering, Koç University, Istanbul, Turkey

**Keywords:** auricular muscle, electrostimulation, Parkinson's disease, walking, motor symptoms, motion capture

## Abstract

It has been demonstrated that intrinsic auricular muscles zone stimulation (IAMZS) can improve the motor symptoms of Parkinson's disease (PD) patients who are examined with the Unified Parkinson's Disease Rating Scale (UPDRS) motor scores. In the present pilot study, using motion capture technology, we aimed to investigate the efficacy of IAMZS compared to medication alone or in combination with medication. Ten PD patients (mean age: 54.8 ± 10.1 years) were enrolled. Each participant participated in three different sessions: sole medication, sole stimulation-20 min of IAMZS, and combined IAMZS (20 min) and medication. Each session was performed on different days but at the same time to be aligned with patients' drug intake. Motion capture recording sessions took place at baseline, 20, 40, and 60 min. Statistical analysis was conducted using one-way repeated measures ANOVA. Bonferroni correction was implemented for pairwise comparisons. The sole medication was ineffective to improve gait-related parameters of stride length, stride velocity, stance, swing, and turning speed. In the sole-stimulation group, pace-related gait parameters were significantly increased at 20 and 40 min. These improvements were observed in stride length at 20 (*p* = 0.0498) and 40 (*p* = 0.03) min, and also in the normalized stride velocity at 40 min (*p*-value = 0.02). Stride velocity also tended to be significant at 20 min (*p* = 0.06) in the sole-stimulation group. Combined IAMZS and medication demonstrated significant improvements in all the time segments for pace-related gait parameters [stride length: 20 min (*p* = 0.04), 40 min (*p* = 0.01), and 60 min (*p* < 0.01); stride velocity: 20 min (*p* < 0.01), 40 min (*p* = 0.01), and 60 min (*p* < 0.01)]. These findings demonstrated the fast action of the IAMZS on PD motor symptoms. Moreover, following the termination of IAMZS, a prolonged improvement in symptoms was observed at 40 min. The combined use of IAMZS with medication showed the most profound improvements. The IAMZS may be particularly useful during medication off periods and may also postpone the long-term side effects of high-dose levodopa. A large scale multicentric trial is required to validate the results obtained from this pilot study.

**Clinical Trial Registration:** www.ClinicalTrials.gov, identifier NCT03907007.

## Introduction

Parkinson's disease (PD) is a neurodegenerative disorder due to dopaminergic neuronal loss at substantia nigra. Hence PD motor symptoms respond well to levodopa medication, levodopa dosages need to be increased gradually as the disease progresses to achieve the same beneficial effects (1–3). In addition, around the tenth year of the disease, the benefits of levodopa for motor symptoms reduce dramatically even with increased dosages, while side effects like dyskinesia become more prominent (1–3). Furthermore, levodopa is also found only to be effective on the locomotion parameters an hour after the oral intake (4). Thus, alternative and adjunct therapies are still needed to overcome these acute and chronic bottlenecks and limitations of existing medications for PD. To date, invasive approaches, like deep brain stimulation, as well as transcranial direct current stimulation, transcranial magnetic stimulation and focused ultrasound-like non-invasive approaches have been used as neuromodulation treatment modalities (5–8). Each of these approaches has its advantages, disadvantages, and limitations. In the context of non-invasive and wearable neuromodulation approaches, the options are limited; hence, more research is needed.

Non-invasive wearable electrostimulation modalities are limited for PD. Transcranial Direct Current Stimulation (tDCS) has been trialed in PD, and the meta-analytic analysis has demonstrated that tDCS could have immediate positive effects on locomotion parameters in PD (9). On the other hand, the effect size was small, and the usability of tDCS has limitations (including the hairy skin) as a wearable device (9). The human auricula has been utilized for numerous non-invasive wearable devices, including auricular vagal nerve stimulators to alleviate multiple clinical conditions (10–13). Although sole auricular vagal nerve stimulation has not been trialed in PD yet, a previous study, Cakmak et al. stimulated the intrinsic auricular muscle zone stimulation (IAMZS) comprising of the auricular vagus nerve in PD patients (14). Stimulation resulted in a moderate-to-large clinical improvement in the clinical motor symptoms (bilaterally) graded by the Unified Parkinson's Disease Rating Scale (UPDRS) in off-state PD patients (14). The improvement in the UPDRS motor scores after electrostimulation using Intrinsic Auricular Muscle Zone (IAMZ) was not observed after placebo or electrical-sham stimulation of muscle free zone of the auricula (14). Furthermore, the dry-needling of IAMZ (but not the sham electrostimulation) also demonstrated a statistically significant but not a clinically meaningful improvement on UPDRS scores as in the electrical stimulation of IAMZ. The latter also emphasized the IAMZ's significant role over muscle-free zones of the auricula.

Besides the UPDRS, as another option for measurement of the effect of treatments on motor symptoms, motion capture technology has been incorporated into PD studies as an absolute objective and comprehensive analysis system for the monitorization of motor symptoms in PD, especially for gait-related locomotion parameters (4, 15–20). The major gait variables like *pace-related parameters* (*stride length, stride velocity*), *dynamic stability-related parameters* (*stance rate, swing rate*), and *turning Speed* have been used in motion capture analyses for PD-related gait changes (4, 15–20). It has been shown that pace-related gait parameters can also be used as an indicator of Hoehn and Yahr stage in PD (20). The beneficial effects of levodopa on pace-related parameters were reported previously (4, 19). In contrast, the responses of dynamic stability-related parameters to levodopa were conflicting in the literature (16–19 vs. 4). Moreover, a motion capture study also indicated that levodopa affects turning velocity in PD (4).

Therefore, in the present study, we aimed to investigate the potential effects of IAMZS on locomotion parameters in PD patients. In this context, a motion tracking system is incorporated to eliminate the potential human (physician-based) factors that may influence the UPDRS scores. In addition, we also aimed to investigate the effects of *sole IAMZS* vs. *medication combined with IAMZS* in the same cohort in the short term.

## Materials and Methods

All participants provided their written informed consent prior to participation in the study. The study was approved by the Ethics Committee of Koç University, Istanbul, Turkey, and it was carried out in accordance with the ethical principles for medical research involving humans (Declaration of Helsinki).

Koç University Clinical Trials Ethics Committee Approval Number: 2017.078.IRB1.009.

Turkey Ministry of Health: Follow up number – E-301776. ClinicalTrials.gov Identifier: NCT03907007

### Research Participants

Ten participants with a diagnosis of idiopathic PD were enrolled in this cross-over study to participate in sole-stimulation, sole-medication, and combined-stimulation-and-medication sessions. PD diagnoses were made by a neurologist who is an expert in movement disorders using the United Kingdom Parkinson's Disease Society Brain Bank clinical diagnostic criteria (21). Psychiatric evaluations at baseline were conducted by a psychiatrist using a structured clinical interview for DSM-5 (SCID-5). The study was conducted at Koç University Hospital, Istanbul, Turkey.

All patients underwent a detailed neurological and psychological examination at the beginning of the study. The patients were chosen based on the following criteria, Idiopathic Parkinson's disease diagnosis, Hoehn and Yahr stage ≥2, Bradykinesia (UPDRS part III - item 31 ≥2) and the existence of Resting tremor (UPDRS part III - item 20 ≥2) and/or Rigidity (UPDRS part III - item 22 ≥2) and/or Walking disorder (UPDRS part III - item 29 ≥2). Exclusion criteria included: having a cardiac pacemaker, significant depression or other psychiatric disorders, irregular heart/respiration rate, pregnancy, excessive alcohol consumption, cardiovascular disease history, an electro-active prosthesis, brain surgery history and ongoing transcutaneous electrical nerve stimulation/percutaneous electrical nerve stimulation therapy.

### Design of the Study

**Figure 1:**
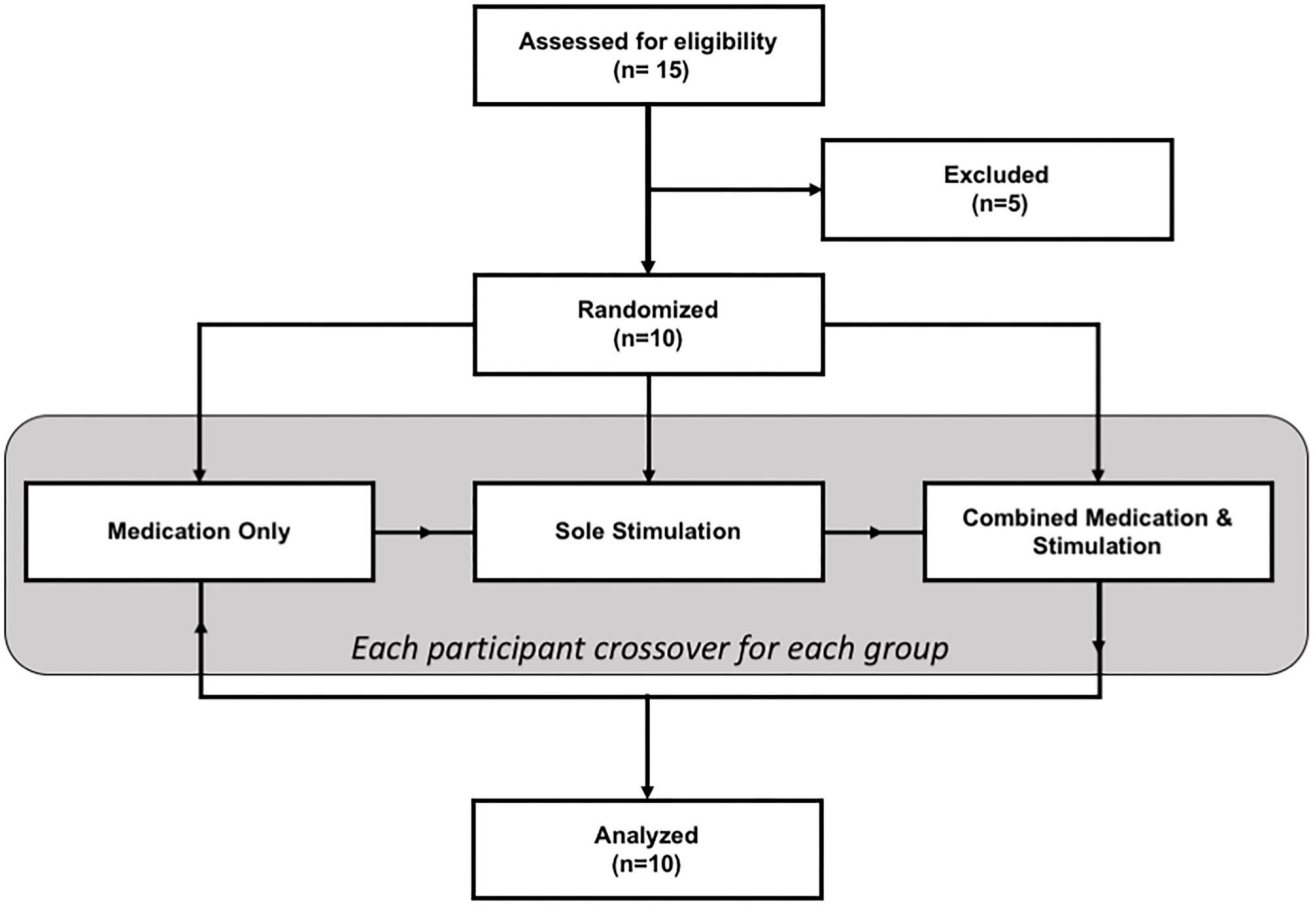
The CONSORT flow chart of the study.

The study had a within-subject design where each patient participated in the study on three different days (one separate day for each session: *sole medication, sole stimulation*, and *combined stimulation and medication*). The sequence of sessions was randomized for each patient. The flowchart of the study is given in Figure 1.

**Figure 2:**
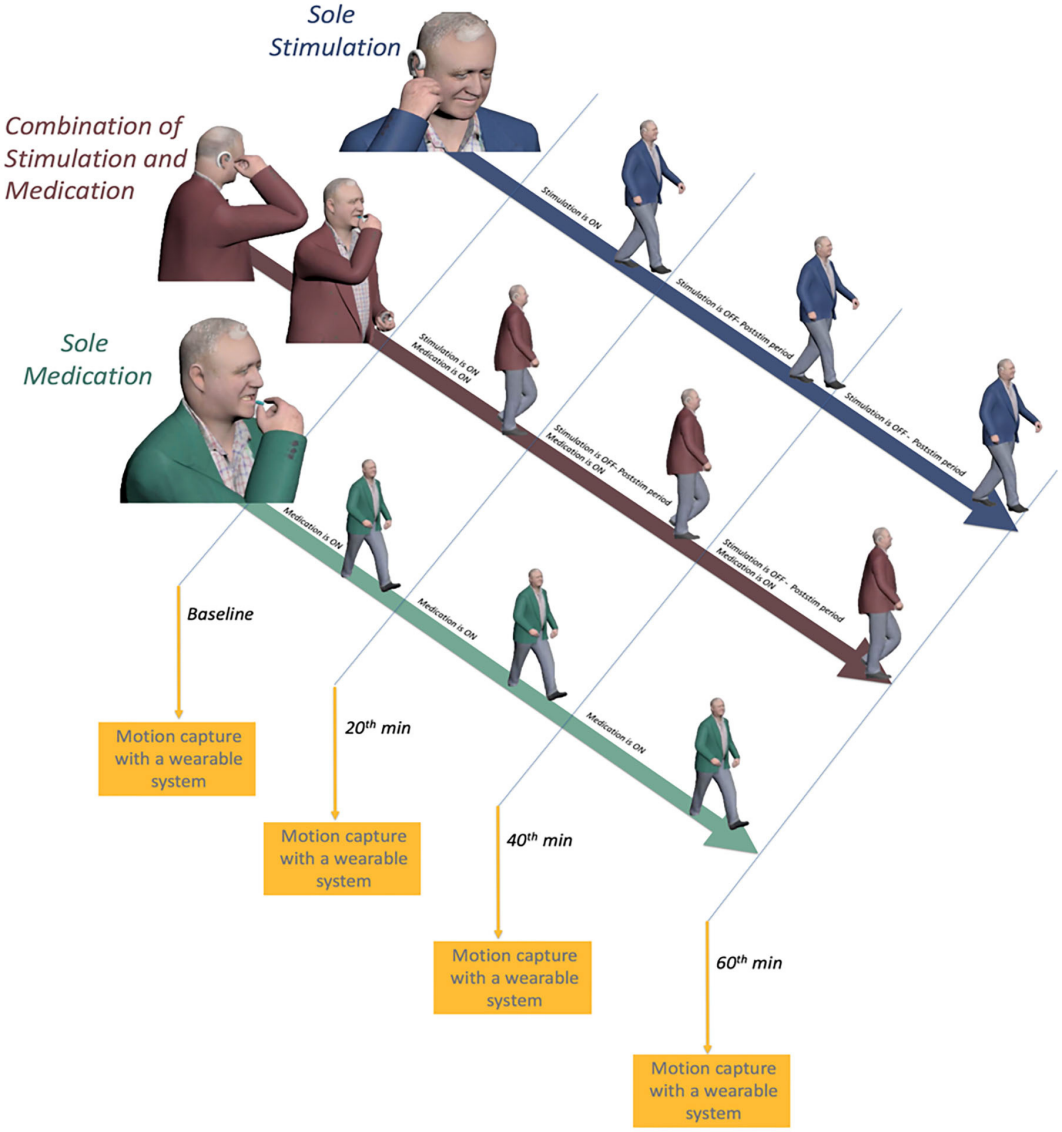
Illustration of the on and off periods of medication and auricular stimulation and motion capture sessions for all three sessions.

During the sole-medication session, IAMZS was not applied. Instead, patients administered their medications at their regularly scheduled time which was aligned with the beginning of the sessions. The purpose of this session was to monitor and observe gait characteristics under their current prescribed medication. Twenty-min-long IAMZS was applied to patients during *sole-stimulation* and *combined-stimulation-and-medication sessions*. The only difference between these two groups was that the patients administered their medication during the c*ombined-stimulation-and-medication* session whereas they skipped one dose of medication (at their regularly scheduled time which was aligned with the beginning of the session) for the *sole-stimulation session*. The details of stimulations and each session are provided in Figure 2.

### Electrostimulation Procedure

**Figure 3:**
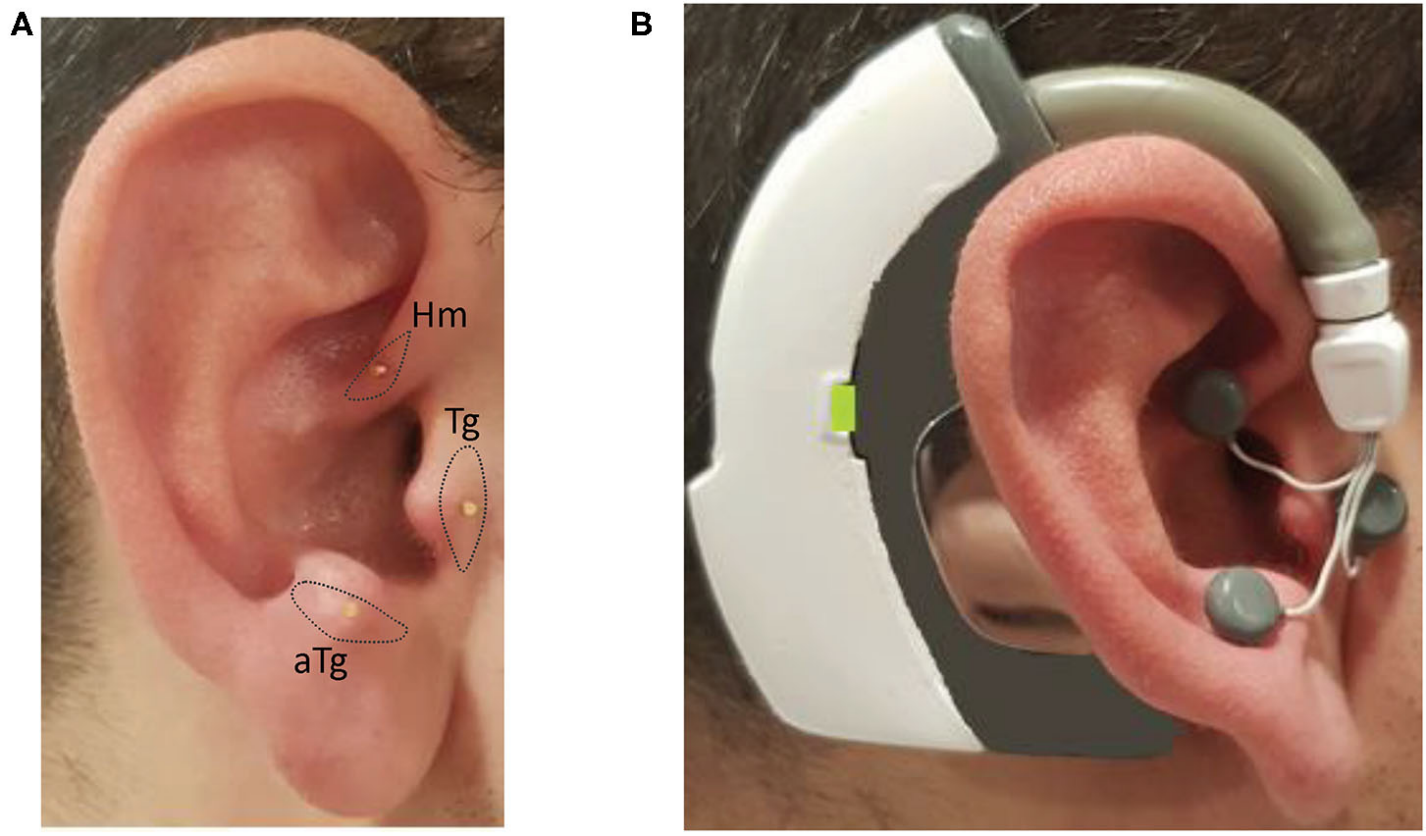
Photos of the inserted needles over the intrinsic auricular muscle zones and the electrical stimulation device worn on the ear with the electrodes attached to needles: **(A)** inserted needles on the intrinsic auricular muscle zones; and **(B)** electrical stimulation device is worn and electrodes attached to needles on the ear.

Twenty-min-long IAMZS (frequency = 130 Hz, pulse width = 100 μs, and intensity under the pain threshold) was performed over the intrinsic auricular muscle zones as described in the previous clinical study (14). The intensity range was between 2.5 and 4.5 V so that it could be perceived as a tingling sensation around the active electrodes. The stimulation intensity was below the pain threshold for all participants. The stimulation was administered unilaterally (ipsilateral to the side of prominent symptoms) as in the previous clinical trial. One of the electrodes that were on the neck in the previous trial (14) to close the electrical circuitry was placed behind the ear in the newly designed version of the wearable stimulator for usability purposes (Figure 3). The wearable electrostimulation device (Figure 3) used in this study is developed at Koç University to be used in this research. It has also been approved to be used in the present study by Koç University Clinical Trials Ethics Committee and Turkey Ministry of Health (follow-up numbers provided in the first section of the methods).

### Clinical Task and Data Measurements

**Figure 4:**
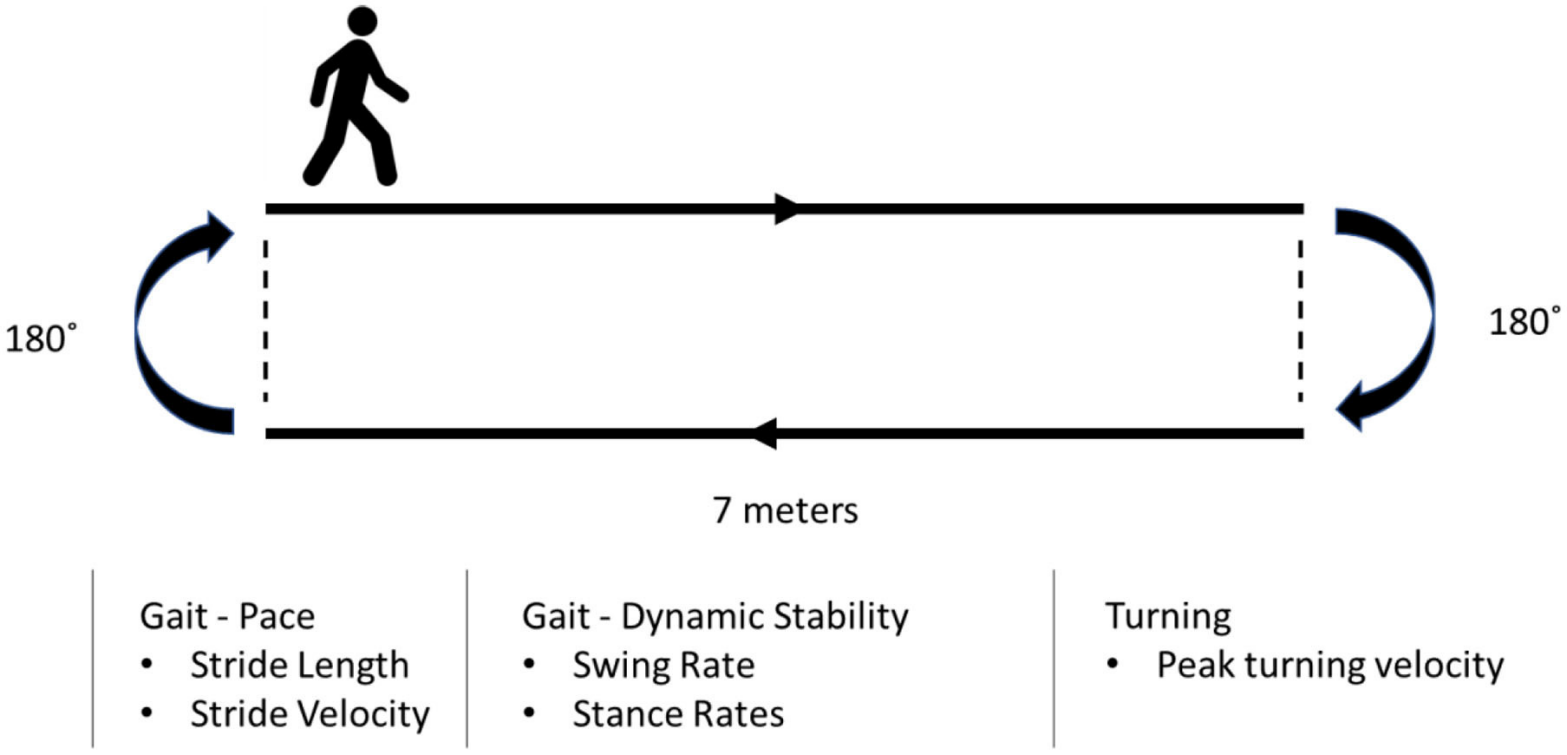
The schematic of the walking task.

Each participant underwent four sequential recording sessions (*baseline, 20 min, 40 min, and 60 min)* during each session throughout the study (Figure 2). Each subject was asked to complete a walking test at a comfortable, self-chosen speed on a flat and obstacle-free surface. The schematic of the walking test is shown in Figure 4. The subjects completed two laps for each recording.

**Figure 5:**
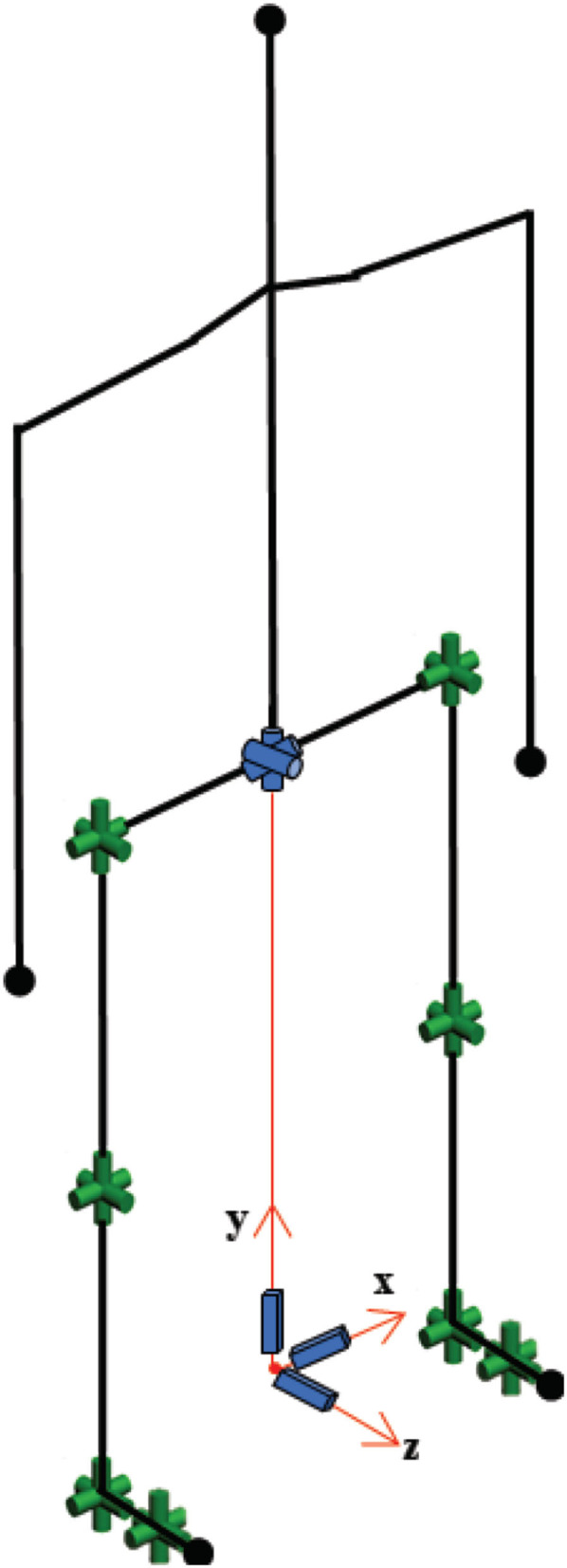
The biomechanical model used for the gait analysis.

Gait data were collected using a commercially available motion capture system, Xsens MVN Link (Xsens Technologies B.V., Enschede, Netherlands), in which inertial-measurement-unit sensors were mounted on the participants' body. The biomechanical model used for gait analysis is provided in Figure 5. The raw data were recorded at 120 Hz. In the first session, segmental link length measurements were taken of the body with the participant in an upright posture (N-pose). The reliability of the wearable motion capture system used in this approach was studied in the literature (20, 22), and its validity has been proven.

### Data Processing

Once the raw data were collected using the motion capture system's dedicated software, MVN Studio, the data were exported to a well-known motion capture file type, BVH (BioVision Hierarchical data). The BVH file consists of relative joint angles of each segment computed from the sensing units and the segmental link lengths measured prior to the recording.

The biomechanical model consisted of 30 degrees-of-freedom as shown in Figure 5. The human model configuration, which includes open-loop kinematic chains, was represented by Denavit-Hartenberg notation (23). Since the objective of this study was to examine the effect of IAMZS on locomotion parameters in patients with PD, the upper body model was simplified and assumed to be a rigid body.

A Butterworth filter (second-order, cut-off frequency = 6 Hz) as implemented previously (22) was applied to the joint angle trajectories represented in the BVH file. The positions of each joint in three-dimensional space throughout the entire recording were calculated by applying the joint trajectories to the forward kinematics in the Denavit-Hartenberg notation in line with previous reports (23, 24).

The model has multiple branches with a global parent branch that is attached to the pelvis formed by the first six joints. Three prismatic joints and three revolute joints modeled in the global parent branch describe the global translation and rotation of the body. The upper body consists of the head, arms, and trunk and is connected to the global parent branch. Since the head, arms, and trunk segment had no effect on the interested locomotion parameters in the present study, and it was assumed to be rigid. The right and left leg segments have hip, knee, ankle, and foot joints.

The entire gait was first segmented into straight walks and turns over the two-lap period as reported previously (25, 26). Then, gait analyses were carried out for all segments. For the straight walk segments, a robust stride detection and identification algorithm were implemented to calculate the spatiotemporal parameters. The algorithm was constructed to detect bilateral heel strike and toe-off instances during straight walking segments. Stride length and time derivative stride velocity were obtained from two consecutive heel strikes. Stride velocity and stride length were normalized with the stature in line with previous motion capture studies in PD (4, 27). The dynamic stability of gait-related parameters (swing and stance rates) were calculated using heel strike and toe-off instances. For the turning segments, the pelvis rotation velocity profile on the transverse plane was obtained.

### Statistical Analysis

One-way ANOVA with repeated measures was used to compare the mean spatiotemporal gait parameters between the baseline measurements and measurements at 20, 40, and 60 min during *sole-medication, sole-stimulation*, and *combined-stimulation-and-medication* sessions, individually. Normality of data distribution was checked with Kolmogorov-Smirnov's test. The greenhouse-Geisser correction was applied for correcting against violation of sphericity, and Bonferroni correction was applied for pairwise comparisons. Unless otherwise stated, data are expressed as mean ± standard deviation. The significance level was set at *p* < 0.05. Statistical analyses were performed using GraphPad Prism version 8.00 (GraphPad Software, La Jolla, CA, USA). Effect sizes for use in ANOVA (η^2^) were also calculated.

Besides the pairwise comparisons, standardized response mean (SRM) calculations were utilized to measure the responsiveness of the IAMZS and medication to the gait measures. The SRM was calculated as the mean change between the corresponding time instances and baseline divided by the standard deviation of the change. An SRM value 0.2–0.5 represents a small, 0.5–0.8 a moderate, and >0.8 a large responsiveness (4).

## Results

Detailed clinical features of the patients are presented in Table 1. The age of the participants was 54.8 ± 10.1 years with a disease duration of 8.4 ± 4.1 years. All the participants were able to complete the walking task without any assistive device throughout the entire study, and no side or adverse effects were reported.

**Table 1:** Participant demographics.

**Patient #**	**1**	**2**	**3**	**4**	**5**	**6**	**7**	**8**	**9**	**10**
Age (years)	46	49	44	58	73	62	48	69	50	49
Sex	F	M	F	M	M	M	M	M	M	F
Stature (m)	1.55	1.87	1.68	1.70	1.68	1.75	1.81	1.75	1.76	1.75
Baseline UPDRS motor score	17	19	15	21	31	15	21	27	25	20
Dominant hand	R	R	R	R	R	R-L	R	R	R	R
Affected side at onset	L	R	R	R	R	R	R	R	L	L
Symptoms at onset	BK	BK	BK	BK	BK	BK	TR	TR	BK	BK
PD type	AR	AR	AR	AR	AR	AR	MX	MX	AR	AR
PD duration (years)	8	17	2	11	8	6	11	6	10	5
Wearing off	Yes	Yes	No	No	No	No	No	Yes	Yes	No
Dyskinesia	No	Yes	No	Yes	Yes	No	No	Yes	Yes	No
H&Y stage	2	2.5	2	2	2	2	2	2.5	2	2
LDED (mg/day)	526	1,697	712	1,072	660	964	300	675	1,297	400

*F, female; M, male; UPDRS, Unified Parkinson's Disease Rating Scale; R, right; L, left; BK, bradykinesia; TR, Tremor; AR, akinetic rigidity; MX, mixed; H&Y, Hoehn and Yahr; LDED, levodopa equivalent dose*.

Once the raw data were processed, spatiotemporal gait parameter values were calculated as explained in section Data Processing. Table 2 shows the calculated gait parameters for all instances of data recordings during the sole-medication, sole-stimulation, and combined-medication-and-stimulation sessions. It should be noted that criteria for normality of data distribution were found to be valid for all dataset.

**Table 2:** Selected gait variables.

**Gait variables**		**Sole medication**				**Sole stimulation**				**Combined medication & stimulation**			
		**Baseline**	**20th min**	**40th min**	**60th min**	**Baseline**	**20th min**	**40th min**	**60th min**	**Baseline**	**20th min**	**40th min**	**60th min**
Pace	Stride length (m/stature)	0.70 (0.07)	0.70 (0.10)	0.75 (0.07)	0.76 (0.07)	0.70 (0.08)	0.73 (0.06)	0.75 (0.06)	0.74 (0.05)	0.71 (0.05)	0.74 (0.05)	0.75 (0.03)	0.77 (0.06)
	Stride velocity (m/s·stature)	0.69 (0.10)	0.70 (0.13)	0.73 (0.10)	0.75 (0.09)	0.69 (0.09)	0.72 (0.09)	0.74 (0.08)	0.73 (0.07)	0.68 (0.07)	0.73 (0.07)	0.74 (0.06)	0.76 (0.08)
Dynamic stability	Stance %	66.20 (3.08)	65.64 (2.55)	66.31 (2.69)	66.10 (2.77)	66.27 (2.74)	66.09 (3.51)	65.73 (3.28)	65.95 (4.01)	66.32 (2.82)	66.48 (3.39)	66.33 (2.29)	66.18 (3.05)
	Swing %	33.80 (3.08)	34.36 (2.55)	33.69 (2.69)	33.90 (2.77)	33.74 (2.74)	33.91 (3.51)	34.27 (3.28)	34.05 (4.01)	33.68 (2.82)	33.52 (3.39)	33.67 (2.29)	33.82 (3.05)
Turning	Turning Speed (deg/s)	175.69 (29.90)	174.39 (27.30)	199.18 (50.15)	187.02 (39.68)	169.62 (23.08)	164.27 (19.05)	170.07 (19.28)	172.89 (20.26)	164.43 (28.37)	170.86 (27.39)	168.50 (24.80)	185.14 (38.63)

*Values are expressed as mean (standard deviation)*.

The first step of the analysis was carried out to compare the baseline characteristics of normalized stride velocity, normalized stride length, stance phase percentage, swing phase percentage, and turning speed variables. As a result of the pairwise comparisons of the baseline characteristics, no significant differences were observed among these variables (Table 3). This indicates that the locomotion capabilities of the subjects were similar over the three days that they participated in the study.

**Table 3:** Pairwise comparisons of baseline characteristics.

	**Sole medication vs. sole stimulation**	**Sole medication vs. combined medication and stimulation**	**Sole stimulation vs. combined medication and stimulation**
Stride length (m/stature)	>0.99	>0.99	>0.99
Stride velocity (m/s·stature)	>0.99	>0.99	>0.99
Stance %	>0.99	>0.99	>0.99
Swing %	>0.99	>0.99	>0.99
Turning speed (deg/s)	0.52	0.66	>0.99

### Sole Medication

During the *sole-medication* session, no significant differences were observed in normalized stride length, normalized stride velocity, swing and stance rates, and peak turning speed among pairwise comparisons (Tables 2, 4). It is worth noting that the magnitudes of the stride length and stride velocity improvements (0.05 ± 0.10 m/stature and 0.06 ± 0.10 m/stature for stride length, 0.04 ± 0.08 m/s/stature and 0.06 ± 0.08 m/s/stature for stride velocity at 40 and 60 min, respectively) observed in the sole-medication group were in line with the previously reported effect of levodopa at 60 min [stride length improvements were reported as 0.02 m/stature for mild cases and 0.04 m/stature for severe cases, and stride velocity improvements were reported as 0.03 m/s/stature for mild cases and 0.05 m/s/stature for severe cases in the previous report (4)]. SRM values revealed that the effect sizes of sole medication to normalized stride length were 0.00, 0.69, and 0.87 at 20, 40, and 60 min, respectively. For the normalized stride velocity, SRM values were 0.09, 0.40, and 0.64. η^2^ values for use in one way ANOVA with repeated measures were obtained as 0.12 and 0.06 for normalized stride length and velocity, respectively.

**Table 4:** Corrected *p*-values of pairwise comparisons of gait variables compared to baseline.

	**Sole medication**			**Sole stimulation**			**Combined medication and stimulation**		
**Gait variables**	**Baseline vs. 20th min**	**Baseline vs. 40th min**	**Baseline vs. 60th min**	**Baseline vs. 20th min**	**Baseline vs. 40th min**	**Baseline vs. 60th min**	**Baseline vs. 20th min**	**Baseline vs. 40th min**	**Baseline vs. 60th min**
Stride length (m/stature)	>0.99	0.47	0.26	***0.05*** (0.0498)	***0.03***	0.15	***0.04***	***0.01***	***<0.01***
Stride velocity (m/s·stature)	>0.99	0.46	0.11	0.06	***0.02***	0.21	***<0.01***	***0.01***	***<0.01***
Stance %	0.40	>0.99	>0.99	>0.99	>0.99	>0.99	>0.99	>0.99	>0.99
Swing %	0.40	>0.99	>0.99	>0.99	>0.99	>0.99	>0.99	>0.99	>0.99
Turning speed (deg/s)	>0.99	0.48	>0.99	>0.99	>0.99	>0.99	0.80	>0.99	0.20

*Bold+italic values represent those which are statistically significant*.

### Sole Stimulation

*Sole-stimulation* resulted in statistically significant improvements in pace-related gait variables (Tables 2, 4). These statistically significant improvements were observed in normalized *stride length* at 20 min (*p* = 0.0498) and 40 min (*p* = 0.03), and also in normalized *stride velocity* at 40 min (*p* = 0.02). *Stride velocity* also tended toward significance at 20 min (*p* = 0.06) in the sole-stimulation group. The significant improvements in the parameters at 20 and 40 min regressed at 60 min (40 min after the termination of the 20-min-long IAMZS), and they were not statistically significant at 60 min in the three-arm pairwise comparisons. However, both parameters were still higher at 60 min than at baseline (both values were 0.04 higher than the baseline values). SRM values revealed that the effect sizes of sole stimulation to normalized stride length were 0.40, 0.65, and 0.57 at 20, 40, and 60 min, respectively. For the normalized stride velocity, SRM values were 0.31, 0.65, and 0.55. η^2^ values for use in one way ANOVA with repeated measures were obtained as 0.08 and 0.07 for normalized stride length and velocity, respectively.

### Combined Application of Stimulation With Medication

**Figure 6:**
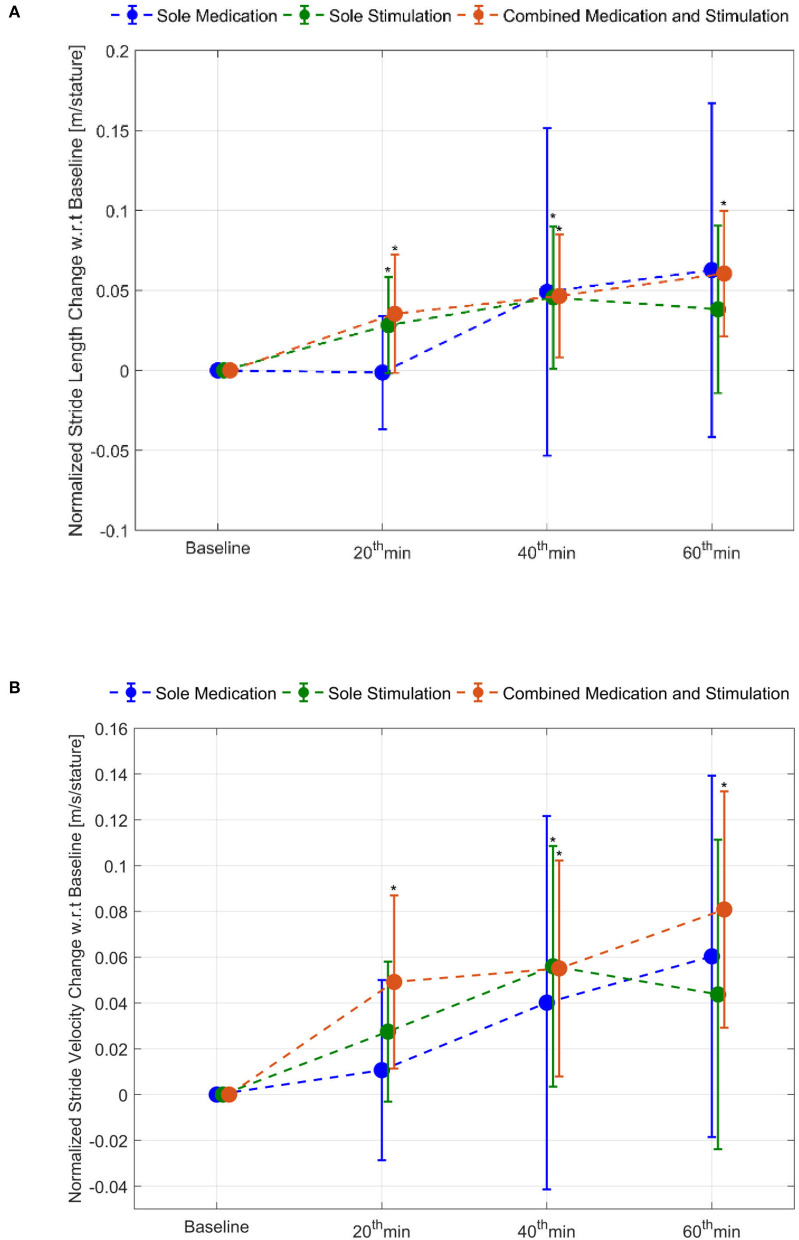
Normalized stride length and stride velocity changes compared to baseline: **(A)** stride length and **(B)** stride velocity. ^*^represents statistically significant changes compared to baseline.

**Figure 7:**
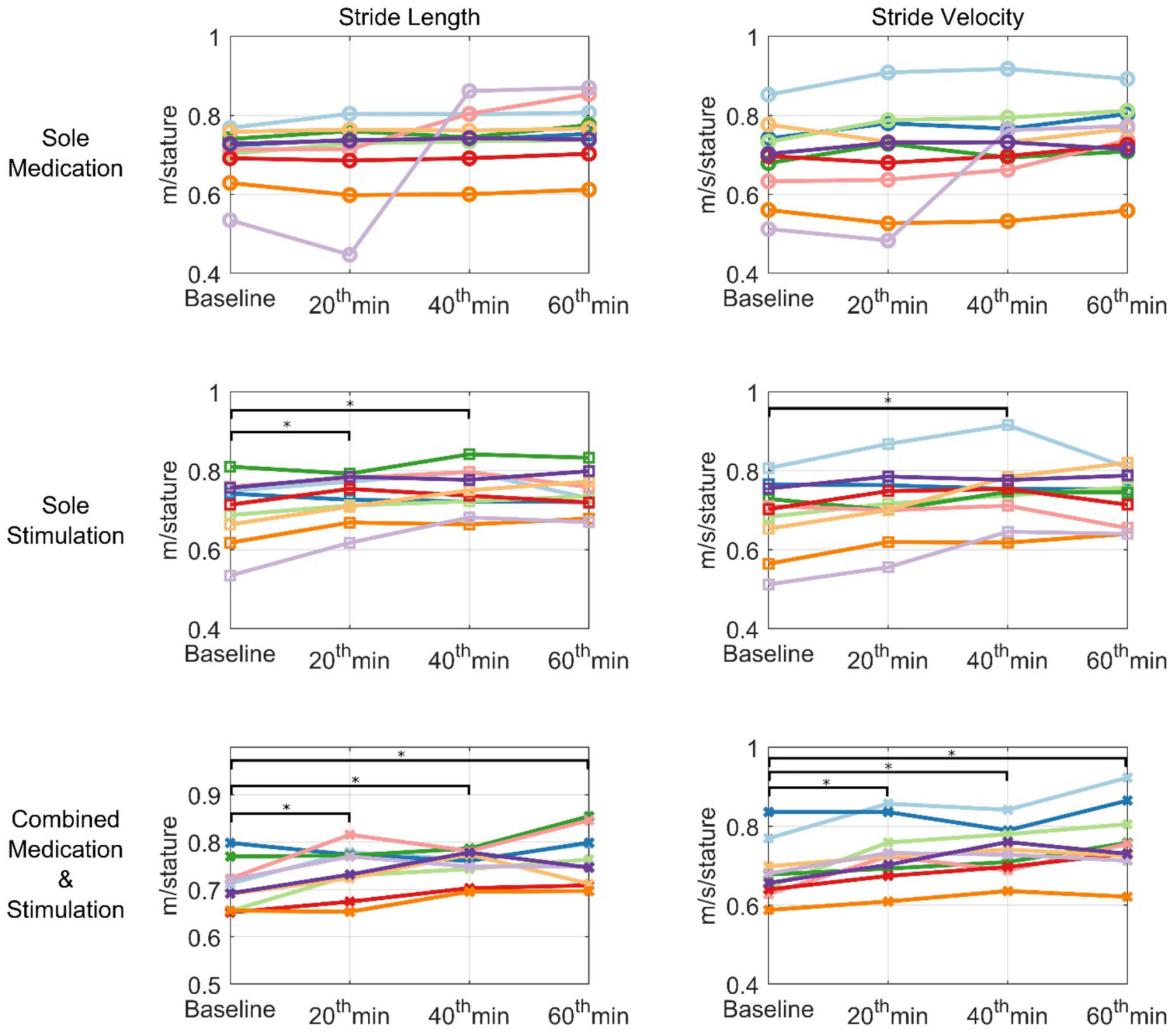
Individual plots of normalized stride velocity and normalized stride length. Each patient is represented using different colors. ^*^represents statistically significant changes compared to baseline.

**Figure 8:**
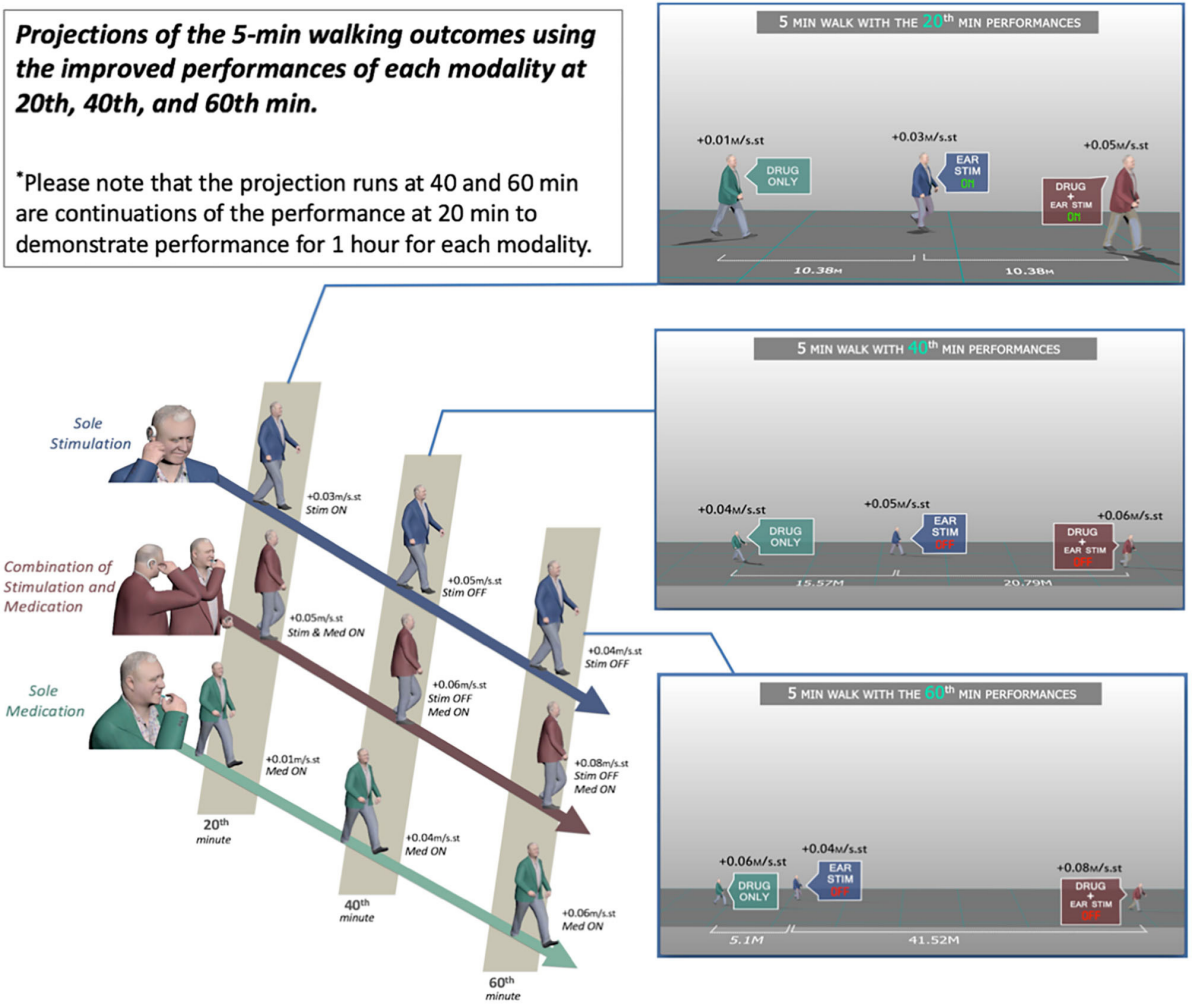
Projections of the 5-min walking outcomes using the improved performances of each modality at 20, 40, and 60 min.

The effect of *combined medication and stimulation* resulted in significant improvements in *normalized stride velocity* and *length* at 20, 40, and 60 min (Tables 2, 4). Normalized stride length and velocity changes compared to baseline for each session can be seen in Figure 6, and the individual plots of normalized stride length and velocity can be seen in Figure 7. The projections for 5 min of walking using the improved stride velocity performances at 20, 40, and 60 min for each group are provided in Figure 8 and as a video file (Supplementary File 1-Video). SRM values revealed that the effect sizes of sole stimulation to normalized stride length were 0.71, 1.13, and 1.15 at 20, 40, and 60 min, respectively. For the normalized stride velocity, SRM values were 0.60, 0.72, and 0.92. η^2^ values for ANOVA were obtained as 0.20 and 0.15 for normalized stride length and velocity, respectively.

## Discussion

Levodopa medication is the gold standard therapeutic modality in PD, however the beneficial effects of oral levodopa on the motor symptoms can appear 1 h after the levodopa intake [(4) and it is also confirmed in the present study motion capture results]. Alternative delivery methods of the levodopa has been utilized to overcome this bottleneck of this 1 h off-period, However these approaches also increase the total amount of levodopa intake which may speed up the narrowing of the therapeutic window of levodopa in the following years and lead to the early onset of side effects. Non-invasive neuromodulation is one of the potential drug-free therapeutic approaches that needs to be investigated. Wearable non-invasive approaches may provide a potential continuous support to PD in their daily routine. In this context, the outcomes of the present pilot study of a wearable electrostimulator demonstrated significant implications that may help the daily life of the PD patients.

Motion capture technology provides a useful tool to demonstrate objective and quantitative changes of gait parameters. Using this tool we demonstrated that the IAMZS has the potential of providing significant beneficial effects of on pace-related gait symptoms in PD in the short term. The stride velocity and stride length parameters of gait can be improved using 20-min-long IAMZS, and the effect was prolonged for 40 min post-stimulation. Forty minutes after the stimulation (at 60 min), the beneficial effects were regressed but still higher than the values at baseline. Compared to sole stimulation, the sole-medication group did not show any statistically significant effect in any time frame of the motion capture analysis (including at 20, 40, and 60 min). Notably, the improvements at 60 min in the pace-related parameters of gait in the sole-medication group were in line with the results of previous motion caption reports, although the improvements were not statistically significant in the present study (4). In contrast, the dynamic stability and turning parameters showed no significant changes during sole-medication, sole-stimulation, and combined-medication-and-stimulation sessions in all time points studied.

Compared to the sole-medication group, the sole-stimulation group demonstrated not only a significant effect but also a faster onset. Oral medications have a latency of up to 90 min to demonstrate symptomatic relief in pace-related gait parameters in PD, and new therapeutic approaches have been tried to overcome this off period of oral medications (28, 29). Inhaler levodopa is one of the approaches to overcome this off period and has been shown to have a faster onset compared to oral medications for PD (28, 29). However, such inhaler options do not eliminate the long-term risks of levodopa usage and the resulting narrow therapeutic window with increased side effects, including dyskinesia (1–3). In this context, the results of the present study also demonstrated a new potential approach to overcome the off periods of oral medications without increasing the daily levodopa dosage and related side effects in the long-term.

The auricula houses six intrinsic auricular muscles (the helicis major and minor, tragicus, anti-tragicus, transverse and oblique muscles) which have both of their origins and insertions on the auricula had reflex responses which can be recorded with electromyography (30, 31). In the present study, the IAMZ zone comprised the 3 of the intrinsic auricular muscles(Tragicus, Anti-tragicus and helicis minor, Figure 3A) on the anterior auricular surface such that the facial nerve is stimulated. In addition, the auricular branch of vagus nerve (32, 33), the auriculotemporal nerve branch of trigeminal nerve (32, 33) and the C2 spinal nerve (32, 33) which are all in the IAMZ are also stimulated. The combined stimulation of these structures, which can all relay to the mesencephalic locomotor region (14, 34–40) are preferred for a profound effect on PD motor symptoms. The tragicus and antitragicus muscles have also been shown to simultaneously contract with the orbicularis oculi muscles (41) as an indirect proof of bilateral cortical control such as in the orbicularis oculi. Furthermore, selective muscle afferent nerve stimulation causes significant activation in motor-related areas compared with cutaneous stimuli (42). Muscle afferent stimulation also evokes more widespread cortical, subcortical, and cerebellar activations than cutaneous afferents (42). Overall IAMZS has greater potential to stimulate the motor movement related networks than the sole sensory stimulation of human auricula. The exact mechanism of action requires further investigation.

C*ombined stimulation and medication* has the most effective outcomes, indicating a synergistic effect of stimulation and medication although significant effects were observed for *sole stimulation*. It can be postulated that combined stimulation and medication may have the potential to achieve the same level of symptomatic improvements in pace-related gait parameters using lower doses of levodopa. If this would be the case, such a combined approach may help to extend the therapeutic window and lower the side effects of long-term levodopa use.

Temporary modulation of blood-brain barrier permeability with stimulation of the sympathetic and parasympathetic system has been documented in the literature (43, 44). The intrinsic auricular muscle zone also comprises the auricular sympathetic and parasympathetic zones (14, 32, 33, 45–47). Therefore, the temporary modulation of blood-brain permeability might be the underlying mechanism of action for the most profound effect that is observed in the *Combined stimulation and medication group*.

In a previous study, we demonstrated a clinically significant effect of IAMZS on UPDRS scores for motor symptoms, and the present study also demonstrated the potential beneficial effects of auricular stimulation using an objective methodology, motion capture technology. Thus, the results presented in the present study are free from clinician bias. Moreover, this was a small-scale, cross-over study with a randomized order of sessions; therefore, the present study did not have a sequence effect or a confounding fatigue factor for particular sessions. However, the Bonferroni correction used in the analysis is a conservative approach and may not be appropriate for small-scale studies. Furthermore, this small-scale cross-over pilot study has other limitations. The results were obtained using a 20-min stimulation period. Longer stimulation periods need to be investigated to clarify the longevity of the post-stimulation period and its potential effect. Changes in gait variability (stride-to-stride fluctuations) during walking is one of the unique markers of gait impairment (48). The short-term measures of gait-related parameters in the present study could not reveal gait variability outcomes, because long-term monitoring is essential to examine gait variability (48). In addition, the present study focused on locomotion with lower limb tracking of PD patients; potential effects on the upper limbs should be investigated. We also did not test participants for the affective changes. The effect of longer stimulations on patients' mood should be investigated further. It has been shown that IAMZS has significant effects in comparison to 3 different controls (auricular placebo, sham electrostimulation of muscle-fee auricular zone and dry needling of the IAMZ) (14). In this context, in the present small cohort pilot study performed we compared the IAMZS to an another treatment (medication) with the aid of an objective monitorization technique(motion capture technique). In future large scale studies, similar control groups can be incorporated into the protocol. Finally the study focused on the acute effects and follow-up sessions were not performed. These aspects should be taken into consideration in future studies.

## Conclusion

In the previous sham- and placebo-controlled trial, IAMZS on PD patients demonstrated clinically significant improvements in UPDRS scores. The present study was the second clinical investigation in which motion capture technology was incorporated to objectively analyze the locomotion parameters for sole stimulation, sole medication, and combined stimulation and medication. The results of this small-scale, cross-over motion capture pilot study in patients with PD emphasized the fast onset of the effects of IAMZS as a sole and as a combined therapeutic approach with medication(with the most profound effect) to improve pace-related gait parameters in the short-term. These results may have significant implications regarding the potential effects on the long-term use of levodopa with better outcomes when combined with IAMZS and for IAMZS as a sole therapeutic approach. A large-scale, multicentric study is needed to validate the positive results obtained in this small scale, pilot study.

## Data Availability Statement

The raw data supporting the conclusions of this article will be made available by the authors, without undue reservation.

## Ethics Statement

The studies involving human participants were reviewed and approved by Koç University Clinical Trials Ethics Committee Approval Number: 2017.078.IRB1.009 ClinicalTrials.gov Identifier: NCT03907007, Turkey Ministry of Health: Follow up number – E-301776. The patients/participants provided their written informed consent to participate in this study.

## Author Contributions

YC: developed the concept. YC, YG-O, and SE: experiment design. YC and BO: designing and performing the stimulation experiments, analyzed data, and wrote the draft. GK, SE, YG-O, OC, HY-E, and EO: performed patient enrolments and/or clinical assessments. YC, HU, SO, and BO: development of hardware, development of software and code writing, and development of analytical tools. BO, YG-O, OC, SO, HY-E, and EO: data collection. YC, BO, GK, SE, YG-O, OC, HY-E, and HU: finalized the main paper. All authors contributed to the article and approved the submitted version.

## Conflict of Interest

YC, BO, SO, and HU have granted and issued patents for the IAMZS devices. Early stage technology, innovation and IP investment, commercialization, and advisory company (Inventram) of Koc Holding & Koc University funded the study and BO's time dedicated to the present study. The remaining authors declare that the research was conducted in the absence of any commercial or financial relationships that could be construed as a potential conflict of interest.
